# Astrovirus-Associated Polioencephalomyelitis in an Alpaca

**DOI:** 10.3390/v13010050

**Published:** 2020-12-30

**Authors:** Leonore Küchler, Isabelle Rüfli, Michel C. Koch, Melanie M. Hierweger, Ronja V. Kauer, Céline L. Boujon, Monika Hilbe, Anna Oevermann, Patrik Zanolari, Torsten Seuberlich, Corinne Gurtner

**Affiliations:** 1 Institute of Veterinary Pathology, Vetsuisse-Faculty, Universitiy of Bern, 3012 Bern, Switzerland; corinne.gurtner@vetsuisse.unibe.ch; 2 Clinic for Ruminants, Vetsuisse-Faculty, University of Bern, 3012 Bern, Switzerland; isabelle.ruefli@vetsuisse.unibe.ch (I.R.); patrik.zanolari@vetsuisse.unibe.ch (P.Z.); 3 Division of Experimental Clinical Research, Vetsuisse-Faculty, University of Bern, 3012 Bern, Switzerland; michelkoch@gmx.ch (M.C.K.); melanie.hierweger@vetsuisse.unibe.ch (M.M.H.); ronja.kauer@gmx.de (R.V.K.); c_boujon@yahoo.fr (C.L.B.); anna.oevermann@vetsuisse.unibe.ch (A.O.); torsten.seuberlich@vetsuisse.unibe.ch (T.S.); 4 Institute for Veterinary Pathology, Vetsuisse-Faculty, University of Zurich, 8006 Zurich, Switzerland; hilbe@vetpath.uzh.ch

**Keywords:** astrovirus, polioencephalomyelitis, alpaca, immunohistochemistry, real-time RT-PCR, next generation sequencing

## Abstract

An 8-year-old alpaca was admitted to the emergency service of the Clinic for Ruminants in Bern due to a reduced general condition and progressive neurological signs. Despite supportive treatment, its condition deteriorated and the animal had to be euthanized. Histopathological analysis revealed a severe non-suppurative polioencephalomyelitis with neuronal necrosis, most likely of viral origin. We detected abundant neuronal labelling with antibodies directed against two different epitopes of Bovine Astrovirus CH13/NeuroS1 (BoAstV-CH13/NeuroS1), which is a common viral agent associated with non-suppurative encephalitis in Swiss cattle. These findings were further verified by detection of viral RNA by use of in-situ hybridization and real-time RT-PCR. Next generation sequencing revealed that the detected virus genome had a pairwise identity of 98.9% to the genome of BoAstV-CH13/NeuroS1. To our knowledge, this is the first report of an astrovirus-associated polioencephalomyelitis in an alpaca. These results point to the possibility of an interspecies transmission of BoAstV-CH13/NeuroS1.

## 1. Introduction

Astroviruses are small, non-enveloped viruses with a non-segmented, single-stranded positive-sense RNA genome which were first identified in 1975 in stool samples of children suffering from diarrhea [1,2]. Since then, they have been found worldwide in humans, especially in infants and children, suffering from gastroenteritis, and are among the most common pathogens to cause juvenile diarrhea [1,2,3,4]. In recent years, several studies have identified astrovirus-associated CNS infections in humans, mink, cattle, sheep, pigs, and a muskox [1,2,5,6,7,8,9,10,11,12,13,14,15,16]. Remarkably, the majority of these neurotropic astroviruses phylogenetically cluster together in the so-called human–mink–ovine (HMO) clade, in which various enterotropic strains are also included [7,8]. Human cases of astrovirus-associated encephalitis are rare and usually associated with immunosuppression, and a systemic spread of the initial enteric infection with subsequent affection of the CNS has been discussed in these patients [1,2,9]. However, more precise mechanisms of neurologic involvement of these viruses still remain unknown.

In cattle, viruses of two distinct genotype species belonging to the HMO clade have been identified in cases of encephalitis, Bovine Astrovirus CH13/NeuroS1 (BoAstV-CH13/NeuroS1) and Bovine Astrovirus CH15 (BoAstV-CH15) [12,17]. To this day, cases of BoAstV-CH13/NeuroS1-associated encephalitis in cattle have been reported in Switzerland, the USA, Canada, Uruguay, Italy, Japan, and the UK [5,11,12,13,18,19,20]. In Switzerland, BoAstV-CH13/NeuroS1 was detected in up to 34% of cattle with non-suppurative encephalitis and is therefore considered an important etiology for viral encephalitis in this species [11]. Up to now, cases of BoAstV-CH13/NeuroS1 appeared to be restricted to cattle. In contrast to BoAstV-CH13/NeuroS1, the strain BoAstV-CH15 was not only identified in cattle, but a virus almost identical to BoAstV-CH15 was detected in several sheep with neurological disease [16,21]. These findings and other phylogenetic studies suggest that certain astroviruses are not host specific and potentially cross species barriers [2,5,6,15,16]. Here, we describe the detection of a neurotropic astrovirus in the central nervous system (CNS) of a neurologically diseased alpaca in Switzerland, which to our knowledge, is the first described case of astrovirus infection in this species.

### Case

In May 2019, an 8-year-old, 84 kg, domestic huacaya alpaca mare was referred to the Clinic for Ruminants at the Vetsuisse-Faculty, University of Bern, because of a reduced general condition, anorexia, trembling, and signs of colic on-going for two days. The day before referral to the clinic, the animal was treated with spasmolytics by the private veterinarian; however, without improvement of the animal’s condition. The alpaca lived in a group with four other alpacas, which were all considered healthy showing no clinical signs. At admission, the alpaca was in a good body condition, but apathetic and standing in a sawhorse stance. A thorough clinical examination revealed reduced gastrointestinal peristalsis and uncoordinated and stiff movements, particularly affecting the hind limbs, with occasional dragging of the toes, and falling to the ground (video sequence of gait abnormalities submitted as Supplementary Materials). The menace response was absent on the left eye, and from time to time the animal showed tremor. A complete blood cell count and blood chemistry were performed and values were as follows: hematology and blood gas analysis were within normal range, biochemistry showed mild hypomagnesemia (0.89 mmol/L, reference 0.90–1.19 mmol/L), hypophosphatemia (1.16 mmol/L, reference 1.39–2.68 mmol/L), elevated creatine kinase (1065 IU, reference 37–108 IU), hyperbilirubinemia (1.8 μmol/L, reference 0–1.7 μmol/L), and mildly increased levels of gamma-glutamyltransferase (35 IU, reference 11–33 IU). The animal was hospitalized, treated with antibiotics (benzylpenicillin, Penicillin-Natrium Streuli, 30,000 IU/kg, iv, Streuli pharma), non-steroidal anti-inflammatory drugs (ketoprofen, Dolovet, 3 mg/kg, iv, Dr. E. Graeub AG), vitamin B supplements (vitamin B1/B6/B12, B-Neuron, 5 mL, sc, Vetoquinol AG), tocopherol (Tocoselenit; 5 mg/kg, sc, Dr. E. Graeub AG), and sodium selenite (Selen-E Vetag, 0.2 mg/kg, sc, MSD animal health GmbH), and rehydrated with sodium-chloride infusions. Clinical exams were performed daily. Over the next three days, the general condition of the alpaca further deteriorated and neurological signs worsened. The animal developed severe proprioceptive deficits, muscle tremor, had difficulties standing up, and showed compulsive movements to the left. A space-occupying mass within the CNS was suspected. Due to the progression of neurological signs and lack of response to treatment, the owner requested euthanasia of the animal due to the poor prognosis. The animal was euthanized with an intravenous injection of pentobarbital (Esconarkon ad us. vet., 150 mg/kg, iv, Streuli Pharma AG) and was subsequently submitted to the Institute of Animal Pathology at the Vetsuisse-Faculty, University of Bern for a post-mortem examination.

## 2. Materials and Methods

### 2.1. Samples

**Figure 1:**
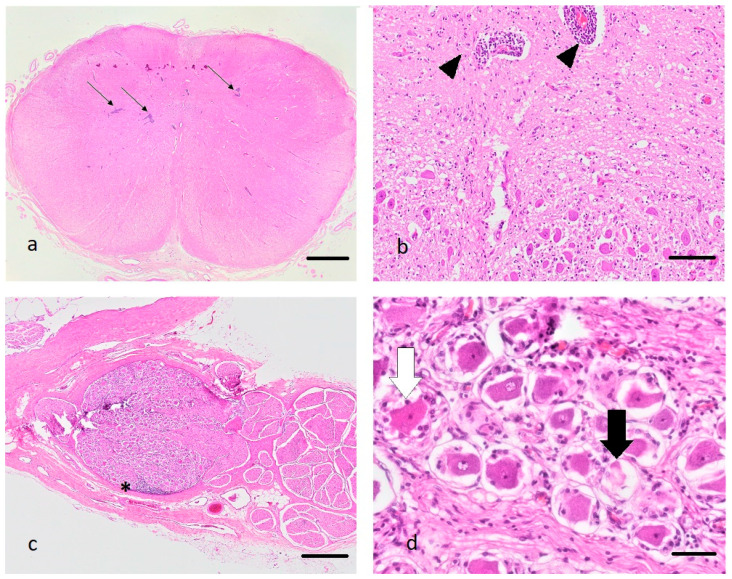
(**a**–**d**): Histopathological changes in the medulla oblongata (**a**,**b**) and the spinal ganglia (**c**,**d**) of the diseased alpaca, hematoxylin and eosin stain. (**a**): Note the presence of multifocal prominent perivascular cuffs (arrows), bar 2 mm. (**b**): Perivascular cuffs consist predominantly of lymphocytes (black arrow heads), bar 100 µm. (**c**): Lymphocytes also infiltrate the spinal ganglia (asterisk), bar 500 µm. (**d**): Some neurons are shrunken and pale (black arrow) or display hypereosinophilia with pyknosis and chromatolysis (white arrow) (neuronal degeneration and necrosis), bar 50 µm.

A full post-mortem examination of the submitted carcass was performed. After gross examination, tissue samples from the brain (medulla oblongata, cerebellum, midbrain, hippocampus, cerebral cortex), spinal cord (cervical, thoracic, lumbar, and sacral spinal cord), heart, liver, uterus, and kidney were collected and placed in 4% formalin. The following day, formalin-fixed tissues were trimmed, embedded in paraffin, and hematoxylin and eosin (HE) stained histological tissue sections were prepared. Slides were then assessed under the light microscope (Figure 1).

### 2.2. Immunohistochemsitry (IHC)

**Figure 2:**
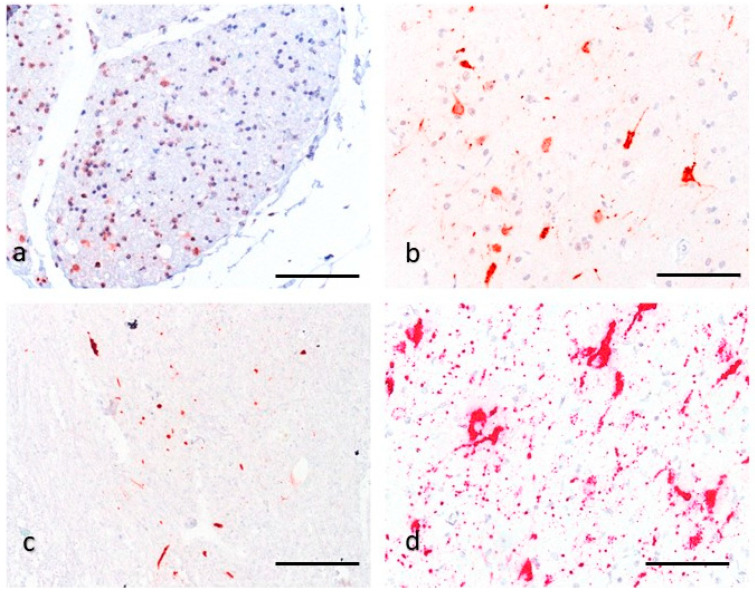
(**a**–**d**): Detection of neurotropic astrovirus infection with immunohistochemistry (IHC) and in-situ hybridization (ISH). (**a**–**b**): IHC for the capsid protein ORF2 of BoAstV-CH13/NeuroS1 of the spinal ganglia (**a**) and the cerebral cortex (**b**) using the polyclonal antiserum ORF2-con. There is strong red granular-like staining of neuronal cells and their extensions, which morphologically appear to be neurons, bar 100 µm. (**c**): IHC for the capsid protein using the polyclonal antiserum CH13-23917. There is multifocal immunolabelling of neuronal cells and their extensions, morphologically compatible with neurons, cervical spinal cord, bar 200 µm. (**d**): ISH; The RNA probe is directed against ORF2 of BoAstV-CH13/NeuroS1. There is massive intracytoplasmic red granular staining of neuronal cells and their extensions, morphologically compatible with neurons, hippocampus, bar 100 µm.

**Figure 3:**
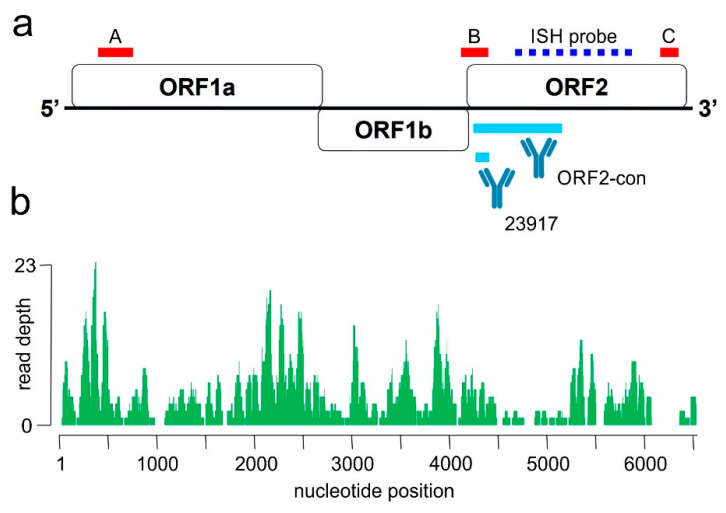
(**a**,**b**): Molecular diagnostic tools for the identification of Bovine Astrovirus CH13/NeuroS1 in the alpaca. (**a**): schematic drawing of the viral genome is presented. Red bars indicate target regions of the BoAstV-CH13/NeuroS1 real-time RT-PCR-A, -B and -C, respectively. The binding side of the RNA probe for in-situ hybridization (ISH) is indicated as a blue dashed line. Polyclonal antibodies for immunohistochemistry were generated against a recombinant protein corresponding to the conserved region (ORF2con) and a 14 amino-acid synthetic peptide (23917) of the capsid protein, encoded by ORF2. (**b**): Results of next generation sequencing of an RNA extract from formalin-fixed and paraffin embedded (FFPE) brain tissue of the affected alpaca. The graph shows the coverage and read depths of the new Bovine Astrovirus CH13 strain along the viral genome.

Two protocols with different antibodies were used for BoAstV-CH13/NeuroS1 detection (Figure 2). The production of hyperimmune sera for each protocol are described in detail elsewhere [8,22]. The first protocol was conducted with the polyclonal antibody CH13-ORF2con, which is directed against the conserved N-terminal half of the capsid precursor protein, encoded by ORF2 of BoAstV-CH13/NeuroS1 [22] (Figure 3a). Formalin-fixed and paraffin embedded (FFPE) tissue samples of the brain (*n* = 5), spinal cord (*n* = 5), heart (*n* = 1), liver (*n* = 1), uterus (*n* = 1), and kidney (*n* = 1) were first deparaffinized, rehydrated, and the endogenous peroxidase reactivity was blocked with a 3% H₂O₂ solution diluted in methanol. Tissue sections were microwaved in Dako Target Retrieval Solution, pH 9 (Agilent, Santa Clara, California, USA). Subsequent protein blocking was performed by using 10% goat serum in phosphate-buffer saline with 0.5% Tween (PBS-T). Each sample was then incubated with a 1:100 dilution of the primary CH13-ORF2con antibody over night at 4 °C. Immunodetection was performed by using the Dako REAL Detection System (Agilent) following the manufacturer’s instructions. The resulting staining was assessed under a light microscope. In the second protocol, the polyclonal antibody CH13-23917 was used, which was generated against a synthetic peptide of the capsid protein of BoAstV-CH13/NeuroS1 6 (Figure 3a). This immunohistochemistry (IHC) was performed on a tissue section of the caudal cervical spinal cord, which had also been tested with the CH13-ORF2con protocol. Apart from microwaving the tissue section in Dako Target Retrieval Solution with pH 6 and an antibody dilution of 1:50 in PBS-T, the same workflow as described above was used for the CH13-23917 protocol. To assess the validity of the IHC, FFPE tissue slides that were tested positive or negative for BoAstV-CH13/NeuroS1 in previous studies served as positive or negative controls, respectively, in every IHC run [12,21,23]. For both IHC protocols, we additionally tested spinal cord and brain tissue sections of a control alpaca, that had shown no neurological signs or histopathological lesions. IHC for detection of rabies was also performed at the Division of Experimental Clinical Research, Vetsuisse-Faculty, University of Bern. IHC for detection of the borna antigen from the hippocampus was performed at the Institute of Veterinary Pathology, Vetsuisse-Faculty, University of Zurich.

### 2.3. Real-Time RT-PCR (RT-qPCR)

RNA was extracted from FFPE tissue of the thoracic spinal cord as described in detail in a paper of Delnatte et al. [24]. Two 20 µm thick sections of FFPE were deparaffinized with xylol and then further processed using the RNeasy FFPE kit (Qiagen, Hilden, Germany) according to the manufacturer’s instructions. For detection and amplification of the RNA extract, we used the recently developed quantitative fluorescent reporter-probe based real-time RT-PCR protocol, which is described in detail in another study [23]. Three different primer-probe combinations were used, targeting the following regions of the BoAstV-CH13/NeuroS1 genome: the 5′end of ORF1a, the center of the genome at the ORF1b-ORF2 interception, and the 3′end of ORF2 (Figure 3a). The respective protocols were designated RT-qPCR CH13-A, -B, and -C. All RT-qPCR reactions were performed by using the AgPath-ID One-Step RT-PCR System (New England Biolabs, Ipswich, MA, USA) according to the manufacturer’s instructions in MicroAmp Optical 96-well reaction plates (Life technologies, Ipswich, MA, USA) in a 7300 Real-time PCR System (Applied Biosystems, Waltham, MA, USA). Fluorescence was measured at the end of each elongation step with the FAM filter and data was analyzed with the Sequence Detection Software (Applied Biosystems, Ver. 1.4).

### 2.4. Next Generation Sequencing (NGS)

RNA was extracted from FFPE tissue of the cranial thoracic spinal cord as described above. Library preparation was done using the TruSeq Stranded Total RNA Kit (Illumina, San Diego, California, USA). The library was quality controlled on a Fragment Analyzer (Advanced Analytical Technologies, Ames, IA, USA) with the High Sensitivity NGS Fragment Analysis Kit (Advanced Analytical Technologies). Next, the library was sequenced on an Illumina NovaSeq 6000 generating 2 × 50 bp reads. Raw reads were quality checked using fastqc (Ver. 0.11.7) and trimmed and filtered with fastp (Ver. 0.12.5, parameters: −l 33 −W 4 −M 15 −5 3 −3 3). The remaining reads were mapped against the host genome (Vicugna_pacos-2.0.1, BioProject PRJNA30567) using STAR (Ver. 2.6.0c, default parameters). Unmapped reads were then mapped to the BoAstV-CH13/NeuroS1 RefSeq entry (accession number NC_024498.1) using bowtie2 (Ver. 2.3.4.1, default parameters). Geneious Prime (Biomatters Ltd., Auckland, New Zealand, Ver. 2019.1.1) was used to create the coverage plot.

### 2.5. In-Situ Hybridization (ISH)

Chromogenic ISH was performed using the RNAscope system (Advanced Cell Diagnostics, Newark, New Jersey, USA) with a BoAstV-CH13/NeuroS1 RNAscope probe directed against ORF2 (Advanced Cell Diagnostics; Cat No. 406921) (Figure 3a). ISH was performed on a FFPE tissue slide of the hippocampus. Staining was carried out with the RNAscope 2.5 Detection Kit Red (Advanced Cell Diagnostics) according to the manufacturer’s instructions. The slide was counterstained in Mayer’s hemalum solution (Merck KGaA, Darmstadt, Germany) and mounted with Aquatex^®^ (Merck KGaA) mounting media. Positive and negative control samples, which were shown to be positive and negative, respectively, for BoAstV-CH13/NeuroS1 in previous studies, were tested at the same time [12,22].

### 2.6. RNA Extraction from Specimen of Conspecifics

Feces, EDTA-blood and nasal swabs of all four remaining alpacas of the herd were taken in August 2019 for RNA extraction. Fecal samples were mixed 1:10 (*w*/*v*) in PBS, vortexed vigorously and centrifuged for 20 min at 4000× *g* at 4 °C. The supernatant was used for RNA extraction. Nasal swabs were transported in universal viral transport medium (BD, Eysins, Switzerland), vortexed and RNA was extracted from the medium. RNA extraction was performed with Trizol LS (Thermo Fisher, Basel, Switzerland) according to the instructions of the provider.

## 3. Results

### 3.1. Gross and Histopathological Findings

On submission, the carcass was in a good nutritional condition. The abdominal cavity contained about 200 mL of serosanguinous fluid (ascites). Both kidneys showed multiple chronic infarcts radiating from the surface to the medulla. The remaining thoracic and abdominal organs were macroscopically within normal limits. Meninges of brain and spinal cord were multifocally congested, but otherwise unremarkable. No evidence of a space occupying mass, as previously suspected, was observed.

On histology, the main lesions were identified within the CNS. Affecting the grey matter of the brain and spinal cord, and also affecting the spinal ganglia, there was a severe inflammatory process, characterized by prominent lymphohistiocytic perivascular cuffs, multifocal degenerated and necrotic neurons, and prominent glial nodules (Figure 1). Within the spinal cord, lymphoplasmacytic infiltrates coalesced, and axons showed degeneration and necrosis characterized by swelling of axons and axon sheaths, axonophagia, and the presence of gitter cells. The most prominent lesions were observed in the caudocervical and thoracic spinal cord. The craniocervical and lumbosacral spinal cord segments were moderately, thalamus, brainstem, and cortex mildly affected by the inflammatory process. Within the liver, hepatocytes diffusely showed mild vacuolization of cytoplasm, interpreted as mild hepatic steatosis. Tissue sections of heart, kidney, and uterus were histologically unremarkable, except for the chronic kidney infarcts. These findings led to the morphologic diagnosis of a chronic, lymphohistiocytic polioencephalomyelitis and ganglioneuritis with gliosis and neuronal necrosis. Based on these lesions, a viral agent was considered the most likely cause of disease.

### 3.2. Results from IHC, ISH, RT-PCR, and NGS

The IHC using the ORF2-con antiserum against BoAstV-CH13/NeuroS1 and the polyclonal antibody CH13-23917 resulted in abundant positive immunostaining within all examined tissue sections of the brain and spinal cord (Figure 2a–c). The positive signal consisted of red granular deposits, mostly within the cytoplasm of cells, which were morphologically compatible with neurons. The localization of positive labelling was in accordance with inflammatory lesions. The IHC of kidney, heart, liver, and uterus remained negative. The validity of each IHC protocol was checked with a negative and positive control sample. In every series of IHC, the positive control showed appropriate immunostaining, whereas the negative control remained negative. Brain and peripheral organ tissue samples from the control alpaca also remained negative. The IHC for detection of rabies and borna antigen remained negative. In conclusion, we considered the alpaca to be immunopositive for both BoAstV-CH13/NeuroS1-ORF2con and BoAstV-CH13/NeuroS1-23917 IHC.

In order to visualize the presence of viral RNA in situ, ISH of the hippocampus was performed. Positive signal consisted of red granular deposits. The probe targeting the ORF2 of BoAstV-CH13/NeuroS1 (Figure 3a) abundantly labelled the cytoplasm of cells with the morphology of neurons (Figure 2d), confirming the presence of BoAstV-CH13/NeuroS1 RNA. Positive control FFPE tissue slides scored simultaneously positive, while positive labelling was absent in negative control slides.

Three months after taking tissue samples at necropsy, RNA extract from FFPE tissue of the thoracic spinal cord was tested by three different BoAstV-CH13/NeuroS1 specific real-time RT-PCR protocols (RT-qPCR CH13-A, -B and -C) targeting different regions of the viral genome (Figure 3a). While the protocols A and B were negative, protocol C, which targets the 3′ end of ORF2, was positive with a Cq of 30. In contrast, the analysis of fecal and nasal swabs and blood samples from the animal’s herd members were negative when tested for astroviruses by real-time RT-PCR.

To further assess the genomic sequence of the detected virus, NGS of total RNA was performed. In total 14′346′488 reads were generated. After quality trimming and filtering, mapping resulted in 496 BoAstV-CH13/NeuroS1 reads covering 83.4% of the BoAstV-CH13/NeuroS1 genome (Figure 3b), with a pairwise identity of 98.9%. In conclusion, the discovered virus is almost identical to BoAstV-CH13/NeuroS1. No other pathological viruses were detected in NGS.

## 4. Discussion

Over the last few years, reports of astrovirus-associated neurological diseases have increased in many species all over the world [5,6,7,8,9,10,11,12,13,14,15,16,17,18,19,20,21,22,23,25]. One of these reported astroviruses is BoAstV-CH13/NeuroS1, which has been frequently identified in cattle in Switzerland suffering from viral encephalitis and is therefore considered an important etiological agent in this species [11,13,14,26]. To our knowledge, BoAstV-CH13/NeuroS1 has not been detected in other animals, and to this day, no astrovirus-infections have been reported in alpacas.

In the present case, an alpaca with progressive neurological signs was euthanized and submitted to our pathology service for further investigation of its illness. Histopathology of the spinal cord and brain revealed a non-suppurative polioencephalomyelitis with neuronal necrosis and gliosis, and a viral etiology was considered to be most likely the cause of disease. Rabies and borna disease are among possible viral agents for non-suppurative encephalitis in alpacas, and were first ruled out by IHC [27,28,29]. When performing IHC on the brain and spinal cord against two different epitopes of BoAstV-CH13/NeuroS1, cells morphologically compatible with neurons were distinctly stained, demonstrating the presence of a virus with similar or identical antigenetic structure to BoAstV-CH13/NeuroS1. Mostly, immunolabelling occurred within areas of pronounced inflammation, supporting a causal link of virus with the disease. However, there was also an absence of immunolabeling in inflamed areas, which could be explained by a potential clearance of the virus in the immune reaction response. Within peripheral organs, no significant histological lesions caused by a viral agent nor immunolabeling by IHC were present, further supporting a particular neurotropism of this virus. The presence of lesions and viral antigen in the spinal ganglia support the notion of neuroinvasion by axonal spread from the periphery, presumably from the gastro-intestinal tract, rather than by systemic infection and/or by the hematogenous route.

Additionally, the ISH on FFPE of the hippocampus with an RNA probe directed against ORF2 of BoAstV-CH13/NeuroS1 confirmed our findings from IHC. The real-time RT-PCR from FFPE tissue of the spinal cord was only positive for the primer-probe combination targeting the 3′end of ORF2, while the other two primer-probe combinations (against the 5′end of ORF1a and the center of the genome at the ORF1b-ORF2 interception) remained negative. The Cq value of 30 for the protocol targeting the 3′end of ORF2 is high, which contradicted our findings from IHC and ISH, in which we clearly demonstrated abundant presence of viral antigen and RNA by targeting this antigenetic region. When mapping reads from NGS on the RNA extract of FFPE tissue against the BoAstV-CH13/NeuroS1 genome, they resulted in coverage of 83.4% of the BoAstV-CH13/NeuroS1 genome with a pairwise identity of 98.9% and demonstrated that the detected virus is almost identical to BoAstV-CH13/NeuroS1. Subsequent analysis of NGS data revealed that binding regions for both, primer and probe for all three real-time RT-PCR protocols, were present within the extracted virus. Even though the amplicons used in each protocol were short, solid results are only provided when the target regions are not fragmented. Therefore, the most likely explanation for the negative results of protocols A and B in the real-time RT-PCR is cross-linking and fragmentation of the RNA in the FFPE tissue of the spinal cord due to exposure to formalin, which can cause RNA extracts to be difficult targets for nucleic acids-based assays [30]. The successful outcome of NGS was likely positively influenced by using reads of short length (50 bp), which were utilized in order to also catch fragmented RNA sequences.

By using different diagnostic approaches, we were able to demonstrate that a virus almost identical to BoAstV-CH13/NeuroS1 was associated with polioencephalomyelitis in an alpaca, which, to this day, is the first report of such an infection in a camelid. The high genomic similarity emphasizes the possibility of an inter-species transmission of neurotropic astroviruses, which has already been postulated for other species [15,16,21]. In the last couple of years, it has been brought to light that cases of neurotropic astrovirus infections involve a variety of different animal species and humans, and investigation into the pathological mechanisms driving disease is a major issue in astrovirus research [1,7,8,9,10,11,15,16,17,18]. Recent preliminary studies on human astroviruses were able to culture human astroviruses within astrocytes in vitro, which is integral in the understanding of astrovirus neuropathogenesis. The production of inflammatory cytokines, as well as the abortive effects of human astroviruses to different neuronal cells, was also demonstrated in this study [31]. However, to this day, apart from immunosuppression, which seems to play an important role in humans, little is known about the factors contributing to the neuroinvasion of astroviruses in other species [1,2,9,17]. Whether the alpaca had suffered from an immunosuppressive condition or disease at some point in life is unknown. Based on our information, the remaining alpacas of the herd are healthy. In cattle, BoAstV-CH13/NeuroS1 was found in fecal samples of clinically healthy animals, suggesting that this virus primarily infects the gastrointestinal tract and that subclinical infected animals serve as a virus reservoir [32]. However, additional retrospective analysis of fecal and nasal swabs as well as blood samples from these alpacas were negative when tested for BoAstV-CH13/NeuroS1 by RT-PCR and to our knowledge, the animal had not been in close contact with cattle or other domestic animals. Ultimately, we were not able to find the source of infection for this particular animal.

Several recent metagenomic studies have identified a huge number of novel astrovirus candidates in mammalian and non-mammalian species. These include rodents, fish, amphibians, reptiles, and insects, suggesting that that the diversity and host range of *Astroviridae* still remains largely unexplored [33,34,35,36]. These hosts species sometimes live in close proximity to humans and livestock and the interspecies jumping of these agents may cause a threat to human and animal health [33]. To assess and predict the zoonotic potential of astrovirus infections, we need a better understanding of infection dynamics, including the shedding and transmission of viruses within and between species. Novel sequencing techniques and bioinformatics workflows are powerful tools to gain more detailed data on phylogenetic relationships of astroviruses from camelids, cattle, humans, and other hosts.

## 5. Conclusions

Our findings demonstrate that neurotropic astroviruses have to be included in differential diagnosis for neurologic diseases in alpacas. Furthermore, we were able to show the close genetic relationship of the detected virus and BoAstV-CH13/NeuroS1 by NGS. These results suggest that BoAstV-CH13/NeuroS1 is not host specific, which has already been postulated for other viruses of this family and can cross the species barrier. Further research should aim to investigate the pathogenesis of this infection and identify factors, which contribute to neuroinvasion and mechanisms of species crossing.

## Data Availability

Raw NGS data are openly available in NCBI SRA (National Center for Biotechnology Information, short read archive) under BioProject ID PRJNA637886.

## Supplementary Materials

The following are available online at https://www.mdpi.com/1999-4915/13/1/50/s1, Video S1: Video sequence of gait abnormalities.
